# Construction of Severe Eosinophilic Asthma Related Competing Endogenous RNA Network by Weighted Gene Co-Expression Network Analysis

**DOI:** 10.3389/fphar.2022.852536

**Published:** 2022-05-11

**Authors:** Haixia Wang, Zeyi Zhang, Yu Ma, Yuanmin Jia, Bin Ma, Junlian Gu, Ou Chen, Shouwei Yue

**Affiliations:** ^1^ School of Nursing and Rehabilitation, Cheeloo College of Medicine, Shandong University, Jinan, China; ^2^ Department of Pediatrics, The Second Hospital of Shandong University, Jinan, China; ^3^ Rehabilitation Center, Qilu Hospital, Cheelo College of Medicine, Shandong University, Jinan, China

**Keywords:** severe eosinophilic asthma, competing endogenous RNA, co-expression network analysis, WGCNA (weighted gene co-expression network analyses), circRNA

## Abstract

**Background:** Currently, disease control in patients with severe eosinophilic asthma is not optimistic. Competing endogenous RNA (ceRNA) networks have been found to play a key role in asthma in recent years. However, it is unclear whether ceRNA networks play an important part in severe eosinophilic asthma.

**Methods:** Firstly, gene expression profiles related to severe eosinophilic asthma were downloaded from the Gene Expression Omnibus (GEO) database. Secondly, the key modules were identified by the weighted gene co-expression network analysis (WGCNA). Thirdly, genes in modules highly associated with severe eosinophilic asthma were selected for further construction of the ceRNA network. Fourthly, Gene Ontology (GO) and Kyoto Encyclopedia of Genes and Genomes (KEGG) enrichment analyses were performed on hub genes. Finally, the results of this study were validated on the GSE143303, GSE137268, and GSE147878 datasets.

**Results:** 22 severe eosinophilic asthmatics and 13 healthy controls were extracted for WGCNA. We found that the genes in the black module (*r* = −0.75, *p* < 0.05) and yellow module (*r* = 0.65, *p* < 0.05) were highly associated with severe eosinophilic asthma. EP300 was discovered to serve the key connecting function in the ceRNA network. Surprisingly, lncRNAs seem to eliminate the role of EP300 in the black module and we discovered that CCT8 and miRNA-mRNA formed a circRNA-miRNA-mRNA network in the yellow module. We found that EP300 and FOXO3 in the black module were regulated by steroid hormones in the enrichment analysis, which were related to the medication used by the patient. Through validation of other datasets, we found that the hub genes in the yellow module were the key genes in the treatment of severe eosinophilic asthma. In particular, RPL17 and HNRNPK might specifically regulate severe eosinophilic asthma.

**Conclusion:** RPL17 and HNRNPK might particularly regulate severe eosinophilic asthma. Our results could be useful to provide potential immunotherapy targets and prognostic markers for severe eosinophilic asthma.

## Introduction

Asthma is an intractable chronic inflammatory illness of the airway caused by complicated genetic and environmental factors, with a wide range of types and intensities of airway inflammation and remodeling (Papi et al., 2018). The majority of patients get standardized medication and care, and their symptoms are successfully managed; nevertheless, 5–10% of asthma patients require high-dose inhaled therapy, yet they have trouble controlling their condition, which is referred to as severe asthma (Lemière et al., 2006; Haselkorn et al., 2009). Severe asthma, which encompasses a wide range of symptoms, is becoming recognized as a highly heterogeneous illness with a wide range of molecular, biochemical, and cellular inflammatory characteristics. Eosinophilic asthma is the most prevalent subtype of severe asthma (Jatakanon et al., 2000; Lemière et al., 2006). The molecular processes underlying the incidence and progression of severe eosinophilic asthma remain unknown. As a result, we need to learn more about them to properly treat severe eosinophilic asthma.

The influence of competitive endogenous RNA (ceRNA) on asthma has recently attracted the interest of researchers as a potential new mechanism for improved asthma therapy. Salmena et al. introduced the ceRNA hypothesis as a unique regulatory mechanism between non-coding RNA (ncRNA) and coding messenger RNA (mRNA) (Thomson and Dinger, 2016). MicroRNA (miRNA)-response elements (MREs), which operate as ceRNAs and play a critical role in different clinical processes, are found in long non-coding RNA (lncRNAs), pseudogene transcripts, circular RNAs (circRNA), viral RNAs, and protein-coding transcripts (Thomson and Dinger, 2016; Fan et al., 2018). With the further development of molecular detection technology, more and more researchers have found that most lncRNAs and circRNAs are involved in the pathogenesis of asthma through the lncRNA-miRNA-mRNA axis and circRNA-miRNA-mRNA axis to regulate the Th1/Th2 balance, M2 macrophage activation, and cytokine (IL-6, IL-13, and IL-17) secretion, respectively (Lee et al., 2009; Han et al., 2019; Huang et al., 2019; Qiu et al., 2019; Shang et al., 2020). The weakness of the previous study is that it focuses mainly on the molecular mechanisms of ceRNA in asthma and not on whether ceRNA plays a role in severe eosinophilic asthma.

Weighted Gene Co-expression analysis (WGCNA) is a systems biology approach that aims to find clusters of genes that are highly correlated with external clinical features to identify potential biomarkers and provide molecular targets for the treatment of disease (Langfelder and Horvath, 2008). Previous researchers have also used the WGCNA approach to explore differential genes in asthma, but previous studies have focused on populations with mild, moderate, and severe asthma and have not explored differential genes between severe eosinophilic asthma and healthy populations (He et al., 2020; Liao et al., 2020). Second, previous studies relied just on WGCNA to identify important genes and did not go on to build ceRNA networks (Kelly et al., 2018; Zhang et al., 2021). Based on this, we used the WGCNA approach to further construct the ceRNA networks to reveal the hidden intrinsic molecular mechanism of severe eosinophilic asthma and provide evidence to support the biologically targeted therapy for severe eosinophilic asthma.

## Materials and Methods

### Data Acquisition and Processing

The GSE143303 (GEO Accession viewer, 2021) was a dataset linked to severe eosinophilic asthma that was retrieved from the Gene Expression Omnibus (GEO) Datasets (https://www.ncbi.nlm.nih.-gov/gds/). GPL10558 was the platform number. Endobronchial biopsies were used to compare 47 samples of distinct inflammatory phenotypes (neutrophilic, eosinophilic, and paucigranulocytic) of severe asthma to 13 healthy controls. For our study, we used endobronchial biopsies from 22 severe eosinophilic asthmatics and 13 healthy controls. 13 healthy controls had a predicted FEV1 of more than 80% and no underlying heart or lung illness. Current smokers were not allowed to participate. The global initiative for asthma (GINA) 2019 (Sánchez-Ovando et al., 2021) defined severe asthma. Patients with severe asthma had no recent history of a clinical chest or upper respiratory tract infection. They also had inhaled corticosteroid (ICS) (>500 μg fluticasone or equivalent per day) or oral corticosteroid (OCS) and were at GINA stages 4–5. The inflammatory phenotypes were defined using the 95th percentile of the differential count and total cell count, and we called it eosinophilic asthma when there were ≥3.50% eosinophils and<71.75% neutrophils (Sánchez-Ovando et al., 2020).

The R Bioconductor package affy was used to normalize raw microarray gene expression data, which was then submitted to multiple quality control techniques. Using annotation information, gene IDs were then mapped to microarray probes. The mean expression value of genes assessed by several probes was determined after probes matching more than one gene were removed from the dataset. Subsequently, the top 5000 genes from the GSE143303 dataset were then screened using the median absolute deviation (MAD) value (Zhao et al., 2021). Following that, we load clinical characteristics data such as inflammatory phenotypes, age, gender, and smoking history (Miller et al., 2010). The *p* value was adjusted by the false discovery rate (FDR) approach. The characteristics of the participants were listed in Supplementary Table S1.

### Network Construction and Consensus Module Detection

WGCNA was able to distinguish genes into multiple clusters, and further investigate the relationship between co-expression modules and clinical phenotypes. The WGCNA package from Bioconductor was used to build co-expression networks for all genes (Langfelder and Horvath, 2008). 1) The hclust function was used to cluster samples and check for outliers; 2) The soft-thresholding power was calculated in the construction of each module using the pickSoftThreshold function of WGCNA, which calculates the scale-free topology fit index for a set of candidate powers ranging from 1 to 20 and provides a suitable power value for network construction. The suitable power was determined if the index value for the reference dataset exceeded 0.85; 3) one-step network building was performed to find co-expression modules, and the limited minimum gene number was set at 50. We assessed the relationship between modules and clinical features. Sample-specific characteristics (e.g., age and smoking) might potentially influence the relationship between gene expression and severe eosinophilic asthma, so logistic regression was used to control for confounding variables in the IBM SPSS Statistics 24.0 software.

### Relating Modules to External Clinical Traits

We were able to find genes with high group significance as well as high module membership in intriguing modules using the gene significance (GS) and module membership (MM) measures. The clinically significant module for severe eosinophilic asthmatics was identified if: |GS| ≥ 0.5 and |MM| ≥ 0.6, the correlation between MM and GS in the module was statistically significant (*p* < 0.05).

The search tool for retrieval of interacting genes (STRING) online database (https://string-db.org/) was an online website that could build a protein-protein interaction network (PPI) based on bioinformatics predictions or biochemical experimental results (STRING, 2021). In this study, the key modules were displayed using the STRING website to create a PPI network detecting gene connections with a threshold of interaction score >0.4. Genes with |GS| > 0.5 and |MM| > 0.6 in the key module were imported to Cytoscape (version 3.8.2). The hub genes were chosen from the top 12 genes.

### Enrichment Analysis

To further visualize the activities of genes in the key module, Gene Ontology (GO) and Kyoto Encyclopedia of Genes and Genomes (KEGG) enrichment analyses were performed on hub genes in the Cytoscape plug-in ClueGo (Bindea et al., 2009). The cutoff threshold was set at a *P* value of less than 0.05. ClueGo classified the signal pathways discovered by enrichment analysis into groups based on functional connection; the same group was colored the same color, and the labels of each group of the most essential terms were color-coded.

### Construction of Competing Endogenous RNA

The RNA Interactome Database (RNAInter) brought together experimentally validated and computationally predicted RNA interactome data from miRTarBase and starBase, including RNA–RNA, and RNA–protein interactions (Kang et al., 2021). The stronger the evidence for a link between the two genes, the closer the confidence score was to 1. The top 12 genes in the black, red, and yellow modules were entered into the RNAInter website respectively to get ceRNA network relationships, and visualization of interacting genes with a confidence score >0.55 in Cytoscape.

### Validation

We validated key genes with three datasets from the GEO database. We retrieved the GSE143303 dataset from patients with severe non-eosinophilic asthma (severe neutrophilic asthma and severe paucigranulocytic asthma) (*n* = 25) and healthy controls (*n* = 13). The aim was to see if the findings were particular to severe asthma or severe eosinophilic asthma. The characteristics of the participants were listed in Supplementary Table S2. We used the GSE147878 dataset to see if the results of this study could be replicated in patients with severe asthma. The GSE147878 dataset was a cross-sectional study from endobronchial biopsies (*n* = 73) and induced sputum (*n* = 44). We extracted the transcriptomic data of bronchoscopic biopsy tissue from severe asthma (*n* = 42) and healthy controls (*n* = 13). The characteristics of the participants were listed in Supplementary Table S3. Finally, the GSE137268 dataset was induced sputum samples from asthmatics and healthy controls. To see if the findings of this study were specific to eosinophilic asthma or severe eosinophilic asthma, we took non-severe eosinophilic asthma patients (controlled and uncontrolled eosinophilic asthma) (*n* = 13) and healthy controls (*n* = 15) in GSE137268. The characteristics of the participants were listed in Supplementary Table S4. The normalized expression values of hub genes were imported into IBM SPSS Statistics 24.0 software, and the differences between the two groups were analyzed using an independent samples t-test.

## Result

### Co-Expression Network Construction

After clustering all samples, we discovered an outlier (sample GSE 4256708) in Supplementary Figure S1. The scale-free topology index exceeded 0.9 when the soft-power β was set to 8 (Supplementary Figure S2). There were 11 modules with sizes ranging from 55 to 1304 genes, labeled 0 through 10 in the order of decreasing size. The number 0 was set aside for genes that did not belong to any of the modules (Supplementary Figure S3).

### Identification of the Clinically Significant Module and Hub Genes

**FIGURE 1:**
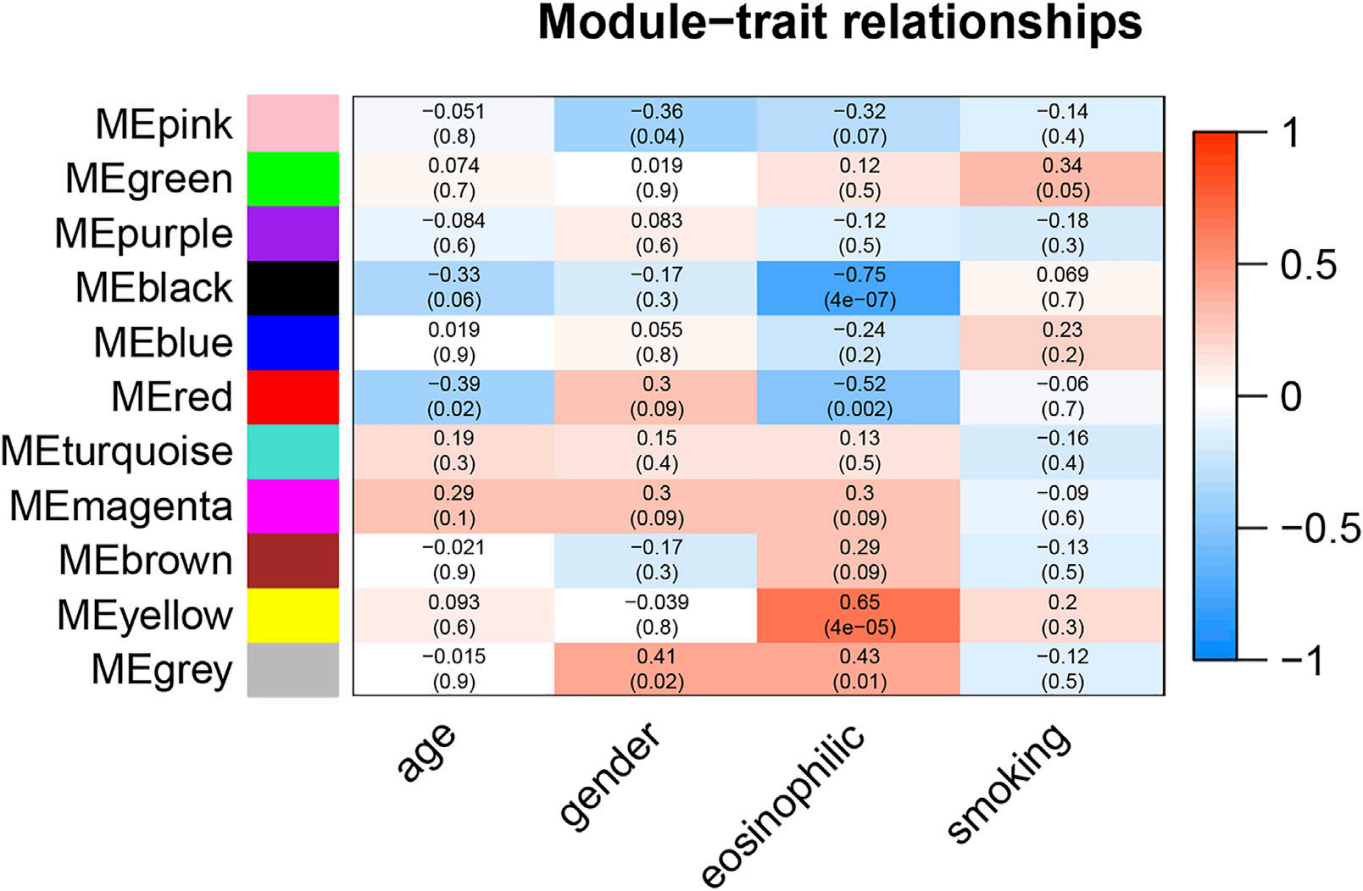
Module-trait associations. Each row corresponds to a module eigengene, column to a clinical trait. The corresponding correlations and *P*-values were presented.

The correlation between module eigengene and clinical features was used to identify module-trait associations in Figure 1. The black and red modules were shown to be adversely associated with severe eosinophilic asthma, with correlations of 0.75 and 0.52, respectively (*p* < 0.05). This indicated that genes in the black and red modules were predominantly downregulated in severe eosinophilic asthmatics. The yellow module was recognized as the positive module with a correlation of 0.65 (*p* < 0.05).

**FIGURE 2:**
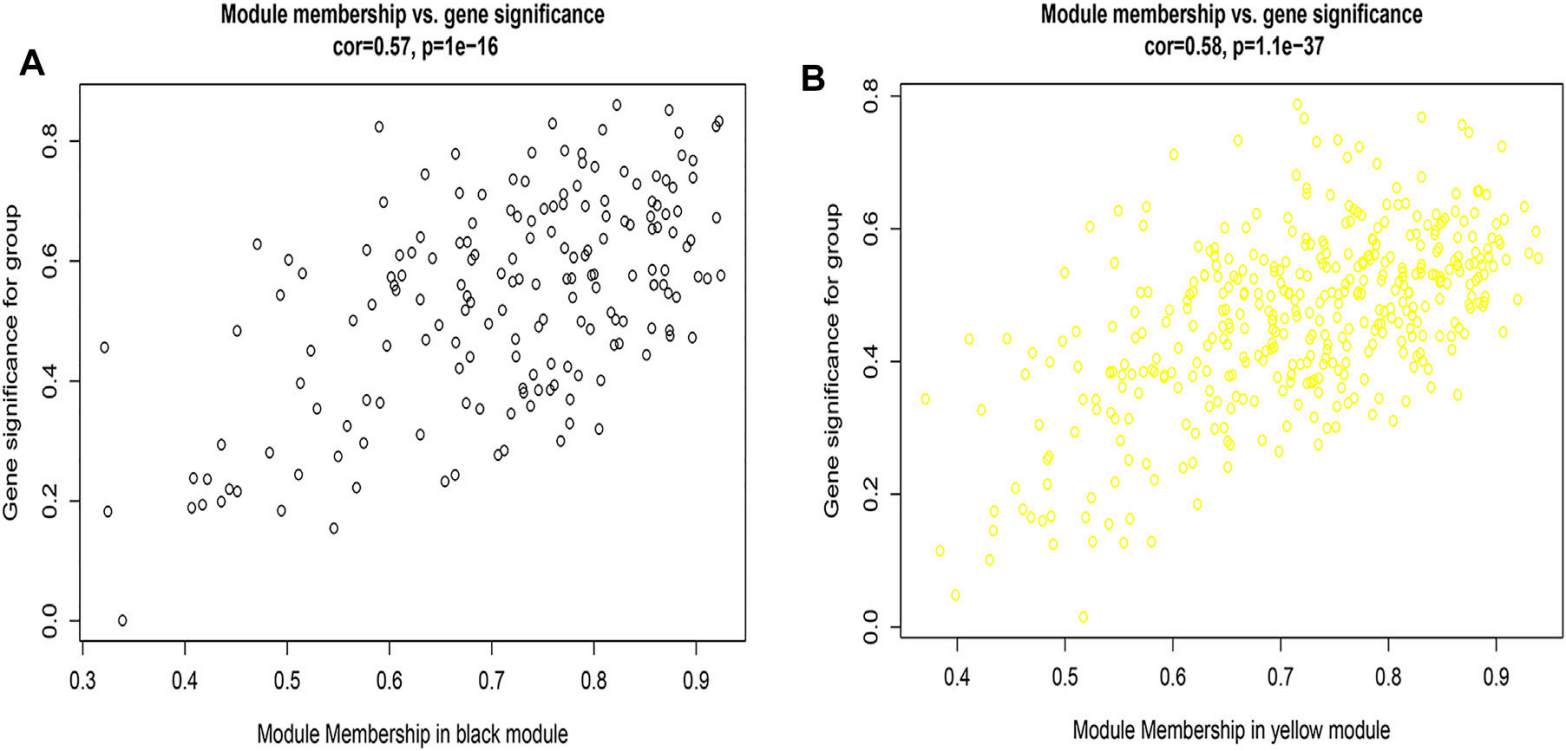
**(A)** A scatterplot of Gene Significance (GS) for eosinophilic vs. Module Membership (MM) in the black module; 2 **(B)** A scatterplot of Gene Significance (GS) for eosinophilic vs. Module Membership (MM) in the yellow module.

**FIGURE 3:**
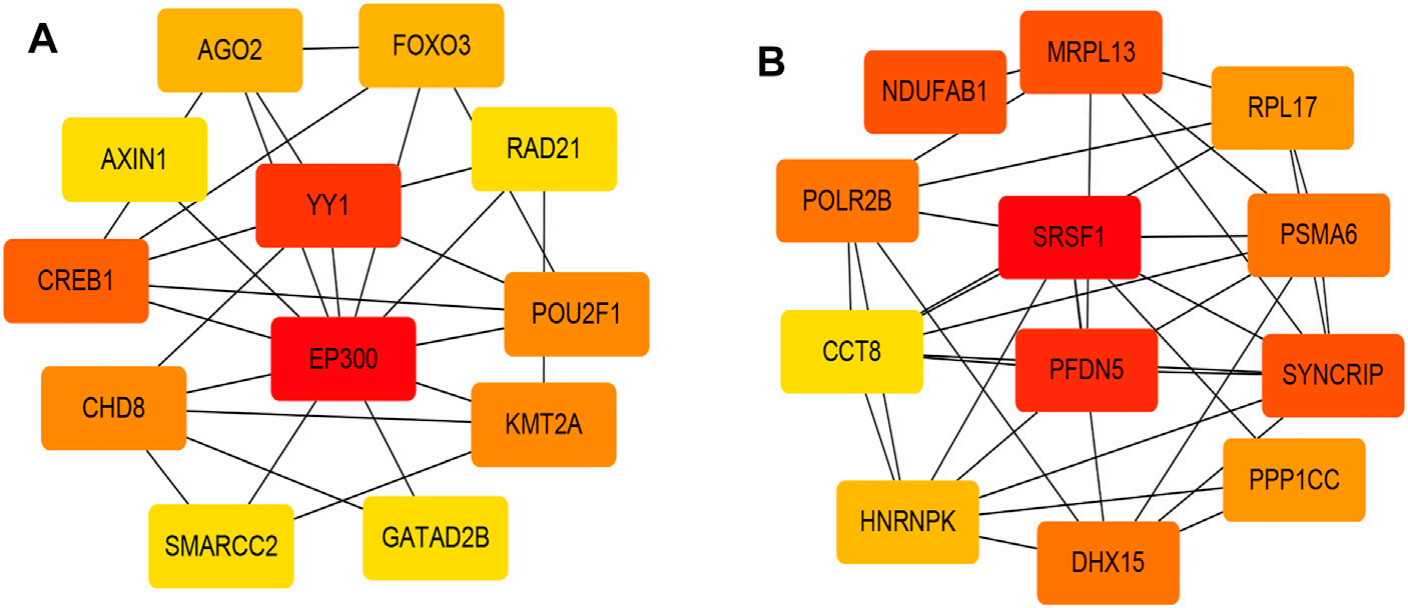
**(A)** The top 12 genes in the black module; **(B)** The top 12 genes in the yellow module. The darker the color, the higher the connectivity of the gene with other genes. The lighter the color, the less connected the gene is to the other genes.

Figures 2A,B, and Supplementary Figure S4 showed that the black, yellow, and red modules had a strong GS-MM correlation (*p* < 0.05), which were identified as the clinically significant module and visualized in STRING (Supplementary Figures S5A–C). In total, 94, 39, and 81 genes with |GS| > 0.5 and |MM| > 0.6 in the black, yellow, and red modules were imported into Cytoscape, respectively, the top 12 genes were filtered as the hub genes in the black, yellow, and red module respectively (Figures 3A,B, and Supplementary Figure S6). Finally, we found that the most connected hub gene was E1A-associated 300-kilodalton protein (EP300), tumor necrosis factor (TNF), and serine arginine-rich splicing factor 1 (SRSF1) in the black, red, and yellow modules.

The hub genes in the red module were strongly associated with the age in Figure 1. After controlling for age, there was no statistically significant difference in the gene for the red module between the two groups of healthy controls and severe eosinophilic asthma (Supplementary Table S5). This indicated that hub genes in the red module might be closely related to the age of the patient.

### CeRNA Network

**FIGURE 4:**
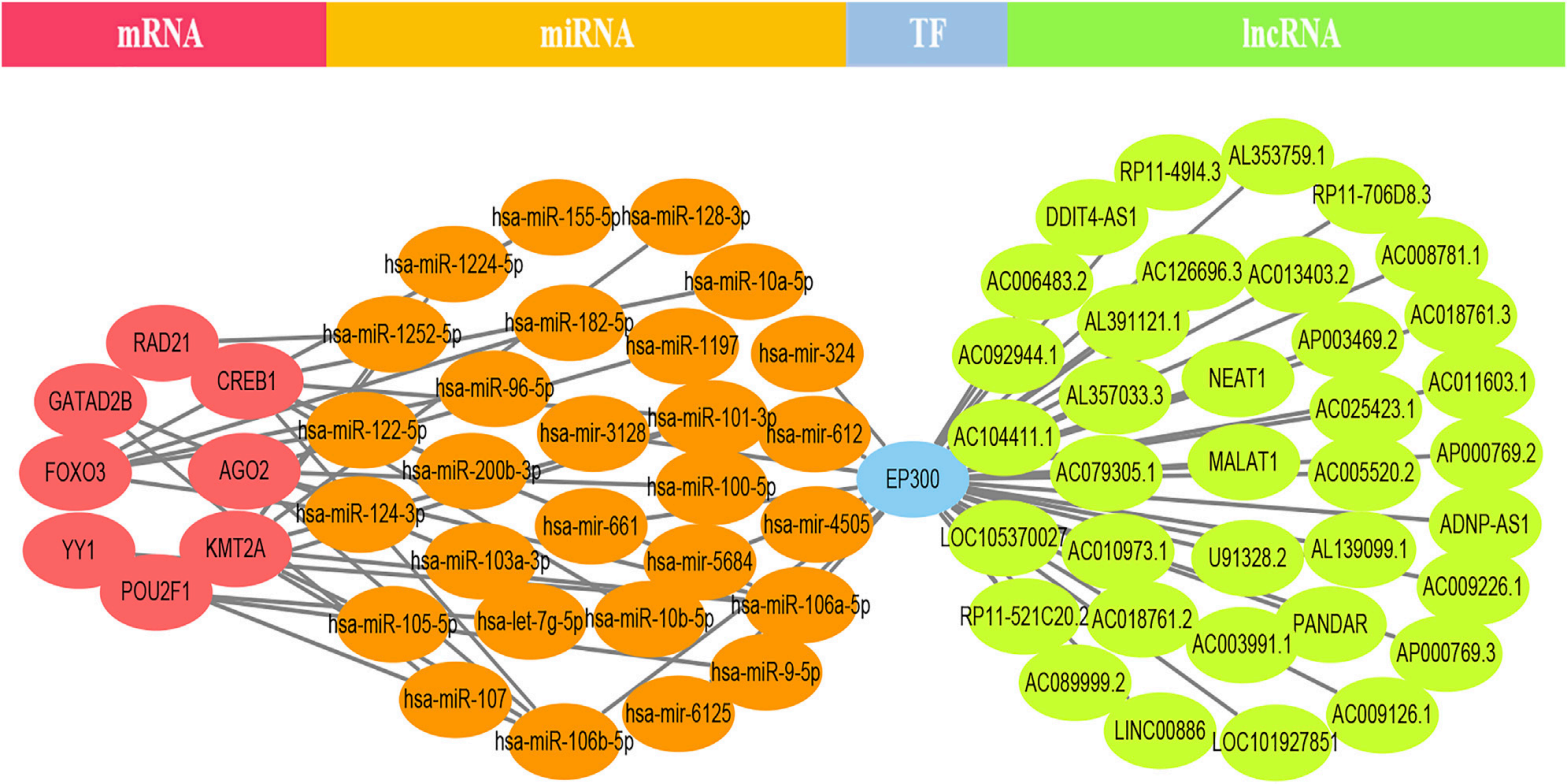
The lncRNA-EP300-miRNA-mRNA ceRNA network of the top 12 genes in the black module. lncRNA would reduce the expression of EP300, which in turn would diminish the role of EP300 as a miRNA sponge and thus cause the down-regulation of mRNA.

**FIGURE 5:**
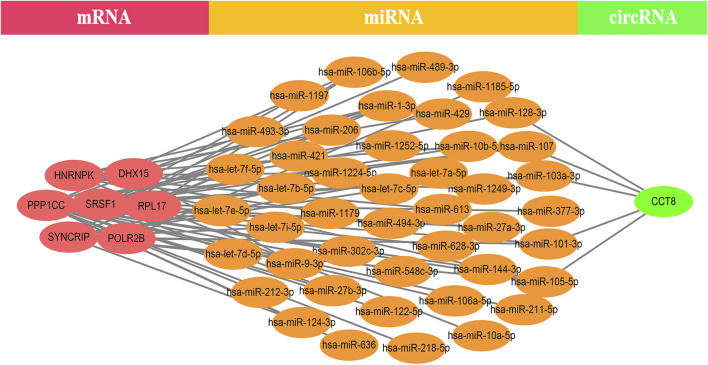
The CCT-8-miRNA-mRNA ceRNA network of the top 12 genes in the yellow module. CCT8 acted as a miRNA sponge and thus cause the up-regulation of mRNA.

EP300 was discovered to serve a key connecting function in the miRNA-mRNA network. Surprisingly, lncRNA would reduce the expression of EP300, which in turn would diminish the role of EP300 as a miRNA sponge and thus cause the down-regulation of mRNA (Figure 4). The hub genes were examined in the same way, and we discovered that CCT8 and miRNA-mRNA formed a circRNA-miRNA-mRNA network. CCT8 acted as a miRNA sponge and thus cause the up-regulation of mRNA (Figure 5). We found that miRNAs such as has-let-7t-5p and has-miR-98-5p bind to TNF, SPI1, and CCR7, limiting their expression in the red module (Supplementary Figure S7).

### Validation

Hub genes in the black module except FOXO3 were down-regulated in the GSE143303 (Supplementary Table S6), which showed hub genes other than FOXO3 might be involved in severe asthma but not particularly regulate severe eosinophilic asthma. FOXO3 may be a key gene in the regulation of eosinophilic asthma or severe eosinophilic asthma. The expression of hub genes other than FOXO3 was decreased in the GSE147878 (Supplementary Table S7), suggesting that hub genes other than FOXO3 might be key genes in the regulation of severe asthma. In addition, the expression of FOXO3 was discovered to be elevated in GSE137268 (Supplementary Table S8), which indicated that FOXO3 was not only implicated in severe eosinophilic asthma but also played a key role in eosinophilic asthma.

We discovered that the expression of hub genes in the yellow module was increased except for Ribosomal protein L17 (RPL17) and Heterogeneous nuclear ribonucleoprotein K (HNRNPK) in the GSE143303 (Supplementary Table S9), which indicated those hub genes other than RPL17 and HNRNPK play a role in severe asthma and do not particularly regulate severe eosinophilic asthma. RPL17 and HNRNPK may be key genes regulating eosinophilic asthma or severe eosinophilic asthma. The GSE147878 dataset further validated that genes other than RPL17 and HNRNPK were key genes regulating severe asthma (Supplementary Table S10). We did not find statistically significant differences in RPL17 and HNRNPK between the healthy control and non-severe eosinophilic asthma groups in the GSE137268 (Supplementary Table S11). Therefore, we speculated that RPL17 and HNRNPK might specifically regulate the specific phenotype of severe eosinophilic asthma.

The hub genes in the red module were all down-regulated in the GSE143303 dataset, but the difference was not statistically significant (Supplementary Table S12).

### Functional Enrichment Analysis

**FIGURE 6:**
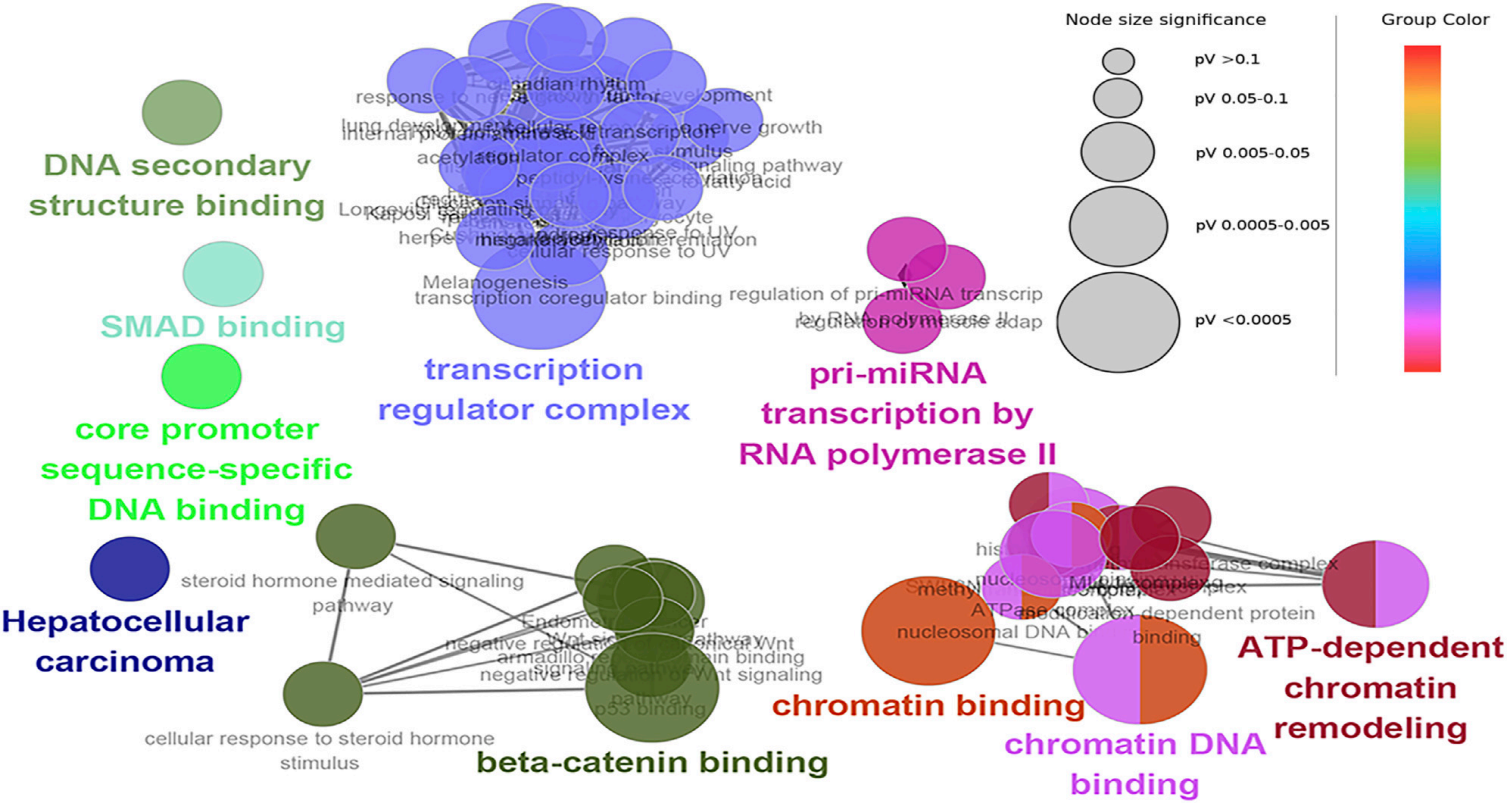
Functional enrichment of top 12 genes in the black module. The signal pathways were discovered by enrichment analysis into groups based on functional connection, the same group was colored the same color, and the labels of each group of the most essential terms were color-coded.

**FIGURE 7:**
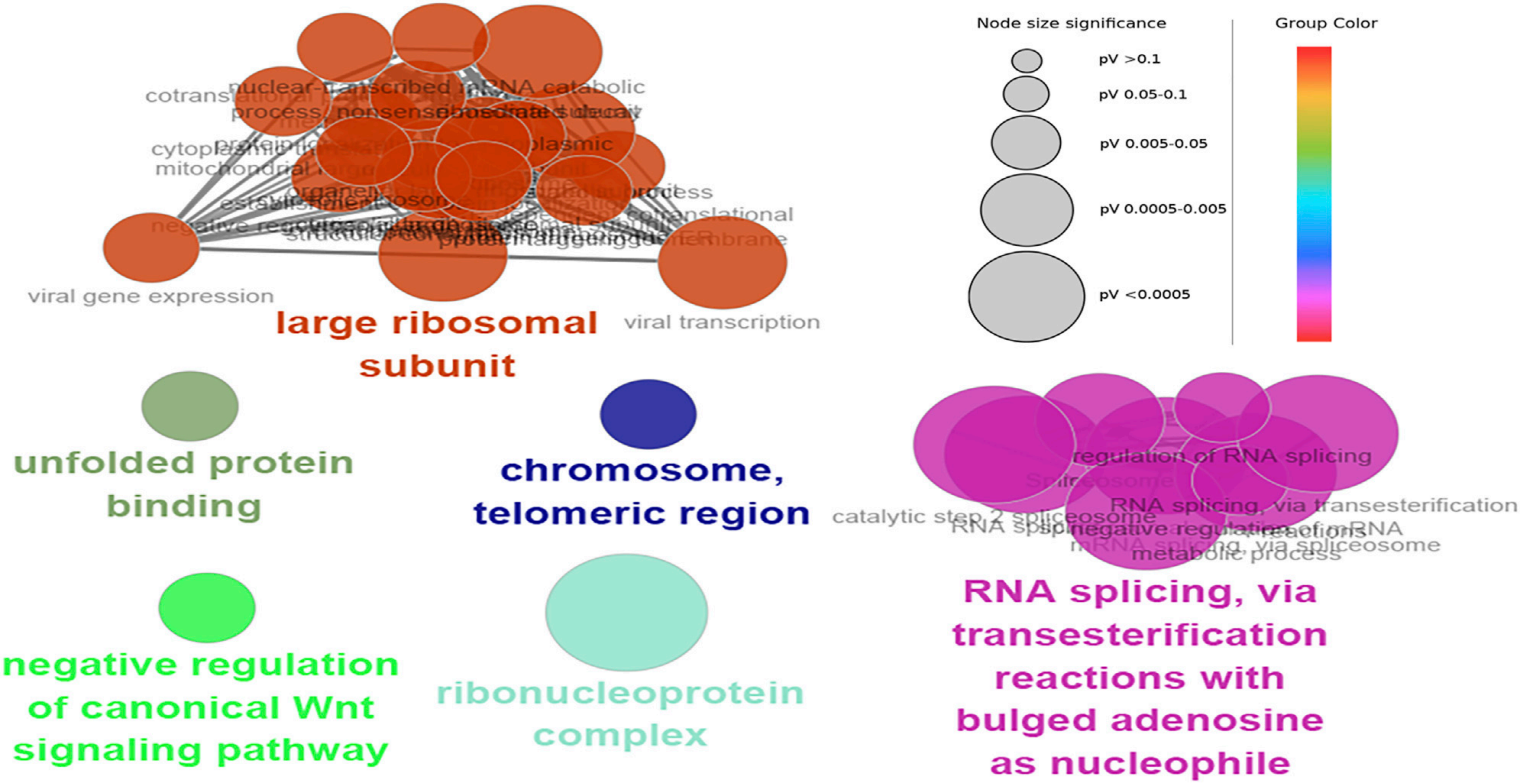
Functional enrichment of top 12 genes in the yellow module. The signal pathways were discovered by enrichment analysis into groups based on functional connection, the same group was colored the same color, and the labels of each group of the most essential terms were color-coded.

The hub genes were mainly involved in the longevity regulating pathway and wnt signaling pathway in the black module. Further investigation revealed that FOXO3 was mainly enriched in pri-miRNA transcription by RNA polymerase II. FOXO3 and EP300 were also involved in chromatin binding, chromatin DNA binding, and beta-catenin binding. Interestingly, beta-catenin binding included steroid hormone mediated signaling pathway and cellular response to steroid hormone stimulus (Figure 6). The hub genes in the yellow module were mainly enriched in the spliceosome and ribosome. Further research demonstrated that CCT8 was mainly involved in unfolded protein binding. SRSF1 and RPL17 were related to the ribonucleoprotein complex. SRSF1 and HNRNPK were involved in RNA splicing, *via* transesterification reactions with bulged adenosine as a nucleophile. KEGG pathway analysis showed that SRSF1 and HNRNPK were mainly enriched in the Spliceosome, while RPL17 was enriched in the Ribosome (Figure 7). The genes in the red module were mainly enriched in viral protein interaction with cytokine and cytokine receptor and B cell receptor signaling pathway. The KEGG pathway of TNF was mainly enriched in Natural killer cell mediated cytotoxicity. GO enrichment analysis showed that TNF, SPI1, and CCR7 were mainly enriched in mononuclear cell migration, lymphocyte activation, regulation of leukocyte activation mononuclear cell migration, and positive regulation of leukocyte migration, negative regulation of immune effector process, and regulation of leukocyte migration (Supplementary Figure S8).

## Discussion

Severe asthma is known to have a variety of phenotypes and endotypes, all of which have a significant impact on patients’ quality of life. The most prevalent phenotype of severe asthma is severe eosinophilic asthma. This is the first research to use WGCNA to create a ceRNA co-expression network in severe eosinophilic asthma. LncRNA-EP300 and CCT8 acting as miRNA sponges played a vital role in severe eosinophilic asthma. In addition, we found that EP300 and FOXO3 in the black module were regulated by steroid hormones, which were related to the medication used by the patient. The genes in the yellow module were the key genes in the treatment of severe eosinophilic asthma, in particular, RPL17 and HNRNPK might specifically regulate severe eosinophilic asthma. This suggests that genes regulating severe eosinophilic asthma and genes regulating severe asthma in the yellow module act in concert in the ceRNA network. We hope that our discoveries will help us better understand and treat severe eosinophilic asthma in the future.

In this study, the median dose of ICS in patients with severe eosinophilic asthma was 1600ug/day, and 5/23 patients received OCS. We found that FOXO3 was involved in cellular response to steroid hormone stimulus. However, the result of validation in GSE137268 showed that FOXO3 was up-regulated and statistically significant in non-severe eosinophilic asthma. FOXO3 is located on chromosome 6q21 and is protein-encoding gene-regulating aging, apoptosis, and tumor. Lützner et al. (2012) found that FOXO3 is a glucocorticoid receptor target that has two functional glucocorticoid responsive regions in its promoter. In the presence of glucocorticoids, FOXO3 stimulates its expression via a positive autoregulatory feedback loop. Yuan et al. (2020) showed that FOXO3 increased in mild asthma patients and decreased in severe patients. This indicated that FOXO3 might be a predictor of sensitivity to glucocorticoids in asthmatics. Non-severe eosinophilic asthma was still sensitive to glucocorticoid medication and had a good treatment outcome. In contrast, patients who were not sensitive to glucocorticoids and had poor treatment outcomes developed severe eosinophilic asthma. Therefore, FOXO3 may be a very significant biomarker for the progression of common eosinophilic asthma to severe eosinophilic asthma.

As we all know, histone modification is a crucial mechanism of epigenetic transcriptional control, histone acetylation (HAT) and histone deacetylases (HDACs) are enzymes that control the acetylation and deacetylation of histones (Cheng et al., 2019). EP300 is a well-known example of endogenous HAT. The levels of EP300 were substantially higher while the levels of histone deacetylases (HDACs) were much lower in asthma. In our study, we found that EP300 was involved in steroid hormone mediated signaling pathways and cellular response to steroid hormone stimulus. Ito et al. (2002) have previously shown that the increased expression of several inflammatory genes in asthma may be due to an increase in HAT activity. In addition, when asthmatic patients were given inhaled steroids, HAT activity was lowered to control levels.

SRSF1 is the archetype member of the SR protein family of splicing regulators (Das and Krainer, 2014). Recent studies demonstrated that SRSF1 is involved in inflammation. Fu et al. (2021) showed that SRSF1 expression was elevated in LPS-induced acute lung injury. However, it is unknown the role of SRSF1 that played in the development and progression of severe eosinophilic asthma. Only a few studies available so far showed that SRSF1 might be a potential biomarker for asthma (Maghsoudloo et al., 2020). The specific mechanism was to be further discovered.

CCT8 is a circRNA and is a member of the TCP-1 chaperone protein (CCT) family of genes. The chaperonin CCT8 controls proteostasis essential for T cell maturation, selection, and function (Oftedal et al., 2021). Current studies have found that CCT8 was overexpressed in cancer, but no study so far has reported any association of CCT8 with asthma. CircRNAs have attracted extensive attention in the pathogenesis of asthma in recent years (Huang et al., 2021). However, the specific roles of these circRNAs in severe asthma were not fully clear.

RPL17 is a member of the L22 family of ribosomal proteins and is the only ribosomal protein that interacts with all six structural domains of 23S rRNA and plays an important role in guiding the proper folding and conformation of 23S rRNA (Pool, 2009). RPL17 was mainly involved in viral gene expression, viral transcription, and cotranslational protein targeting the membrane. There is no evidence that RPL17 plays a role in asthma. However, some studies have explored the relationship between ribosomal proteins and asthma. Dong et al. (2017) showed that ribosomal protein S3 (RPS3) siRNA lessened HDM-induced airway mucus hypersecretion, cytokine production, and serum IgE elevation. Future studies are yet to be required to further explore the connection between RPL17 and severe eosinophilic asthma.

HNRNPK is a DNA/RNA-binding protein and regulates a wide range of biological processes and disease pathogenesis (Wang et al., 2020). The mechanism of HNRNPK involvement in severe eosinophilic asthma is currently unclear. Meng et al. (2021) found that HNRNPK acted downstream of TNFα-TNFR2 signaling, and was involved in the inflammatory response. Additional studies will be required to further explore the relationship between HNRNPK and severe eosinophilic asthma.

After adjusting for age, the expression of genes in the red module was not significantly different between the two groups. The genes in the red module were found to be mainly involved in inflammatory and immune response processes in the enrichment analysis. Increased levels of circulating cytokines and proinflammatory indicators including IL-6, IL-1, and TNF are linked to aging (Michaud et al., 2013). Previous studies have found that both TNF, SPI1, and CCR7 were up-regulated in severe asthma (Wittwer et al., 2006; Niessen et al., 2021; Wang et al., 2021). In addition, Xing et al. (2002) found dexamethasone resulted in significant inhibition of pro-inflammatory cytokines, T-cell stimulation, chemokines, and chemokine receptors. We speculated that the downregulation of hub genes in the red module may be due to differences in the age of the participants and the use of ICS and OCS.

Although the findings of our study have important clinical implications, the limitations must also be noted. First, we did not find a dataset that could validate the relationship between lncRNAs like MALAT1 and NEAT1 and severe eosinophilic asthma. Future studies are therefore expected to deeply explore the relationship between lncRNAs and severe eosinophilic asthma. Second, we could not obtain the dose of ICS and OCS taken by each patient with severe eosinophilic asthma, so we could not control the effect of drug use on the results. However, we also found the effect of drug use on the results by other methods such as dataset validation and enrichment analysis. At the same time, we also identified some genes in the black and red modules that were regulated when conventional drugs were used to treat severe eosinophilic asthma, and potential regulatory genes in the yellow modules of severe eosinophilic asthma that remain uncontrolled by drug therapy, which provided evidence for subsequent targeted and precise treatment of severe eosinophilic asthma.

## Conclusion

In conclusion, our study reported that the ceRNA network played an essential and significant role in severe eosinophilic asthma. The hub genes in the yellow module were the key genes in the treatment of severe eosinophilic asthma. In particular, RPL17 and HNRNPK might specifically regulate severe eosinophilic asthma. Our results could be useful to provide potential immunotherapy targets and prognostic markers for severe eosinophilic asthmatics patients. Further mechanistic studies were required to validate and elucidate this result.

## Data Availability

Publicly available datasets were analyzed in this study. The names of the repository/repositories and accession number(s) can be found in the article/Supplementary Material.

## Abbreviations

ceRNA, competing endogenous RNA; circRNA, circular RNAs; EP300, E1A-associated 300-kilodalton protein; FDR, false discovery rate; GEO, gene expression omnibus; GINA, global initiative for asthma; GO, gene ontology; GS, gene significance; HAT, histone acetylation; HDACs, histone deacetylases; KEGG, kyoto encyclopedia of genes and genomes; lncRNAs, long non-coding RNA; MAD, median absolute deviation; miRNA, viral RNAs, microRNA; MM, module membership; MREs, microRNA response elements; mRNA, messenger RNA; ncRNA, non-coding RNA; PBMC, peripheral blood mononuclear cells; PPI, protein-protein interaction network; RNAInter, RNA interactome database; RPS3, ribosomal protein S3; SRSF1, serine arginine-rich splicing factor 1; STRING, search tool for the retrieval of interacting genes; TFs, transcription factors; WGCNA, weighted gene co-expression analysis.
