# Supplementary material for: Construction of Severe Eosinophilic Asthma Related Competing Endogenous RNA Network by Weighted Gene Co-Expression Network Analysis
Source: Front Pharmacol. 2022 May 11;13:852536. doi: 10.3389/fphar.2022.852536 (PMC9130708; doi:10.3389/fphar.2022.852536)
Supplement: Supplementary file 1 [file DataSheet1.docx]

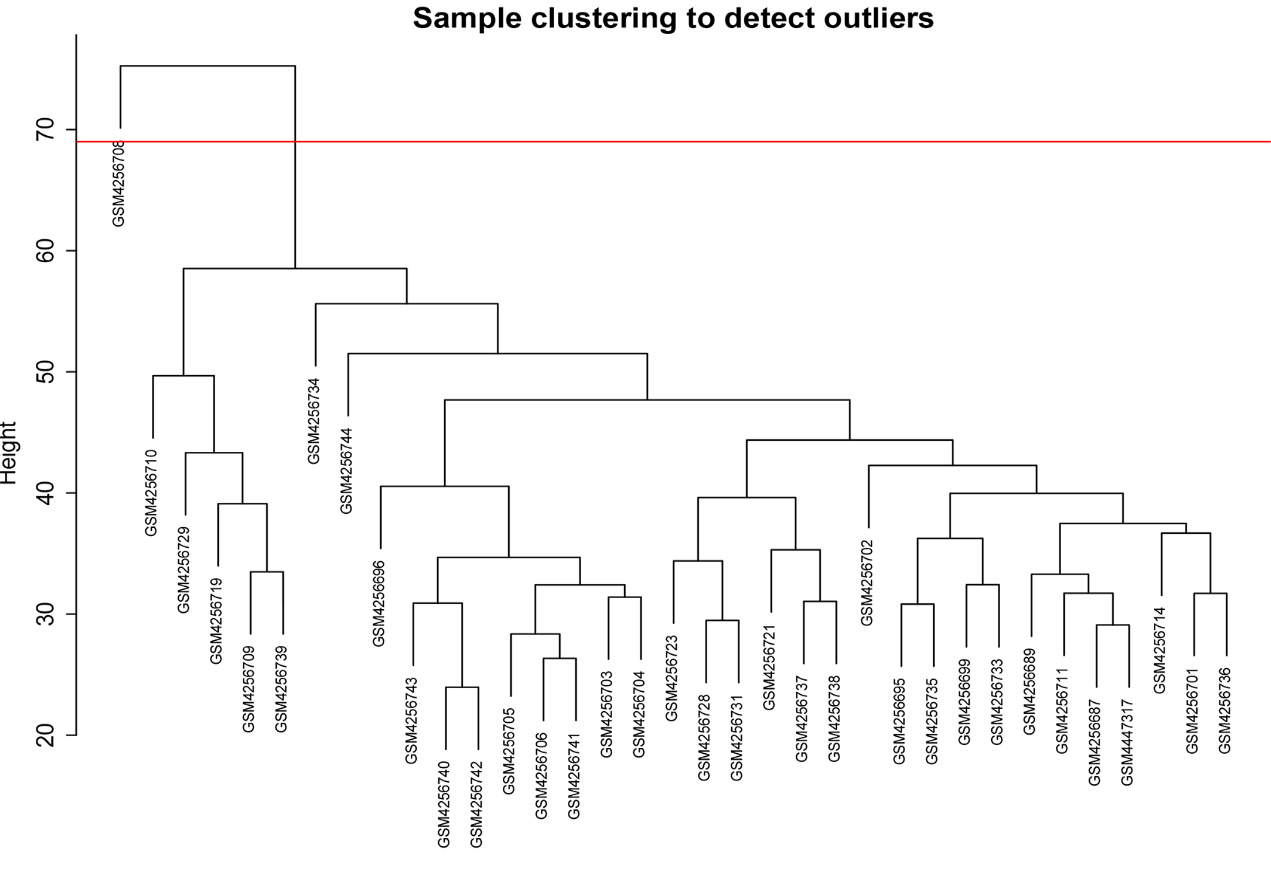


Figure S1: Clustering dendrogram of samples based on their Euclidean distance. Participant GSE4256708 had severe eosinophilic asthma, a 57-year-old female with no smoking history, and an Asthma Control Questionnaire score of 2.3.


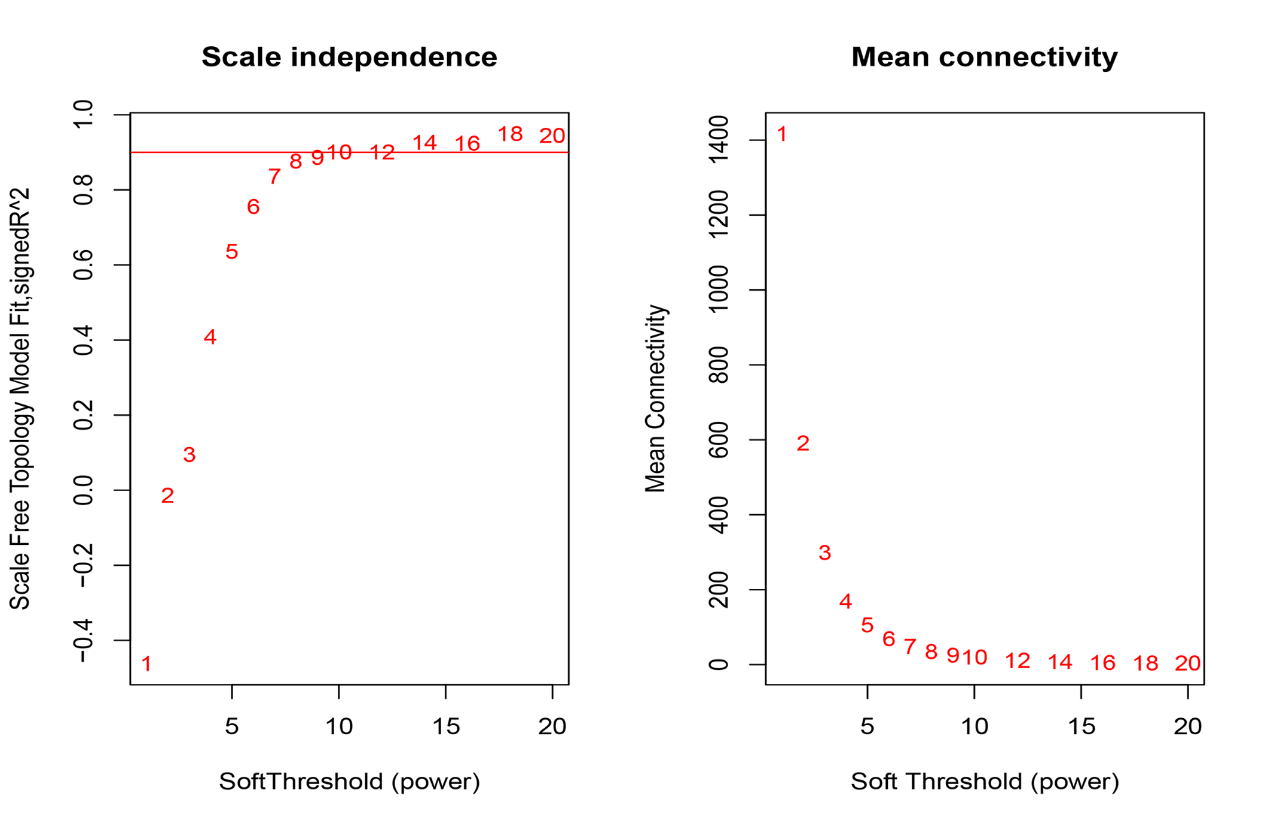


Figure S2: Analysis of network topology for various soft-thresholding powers. With a scale-free topological criterion >0.9, 8 was chosen as the fittest power value.


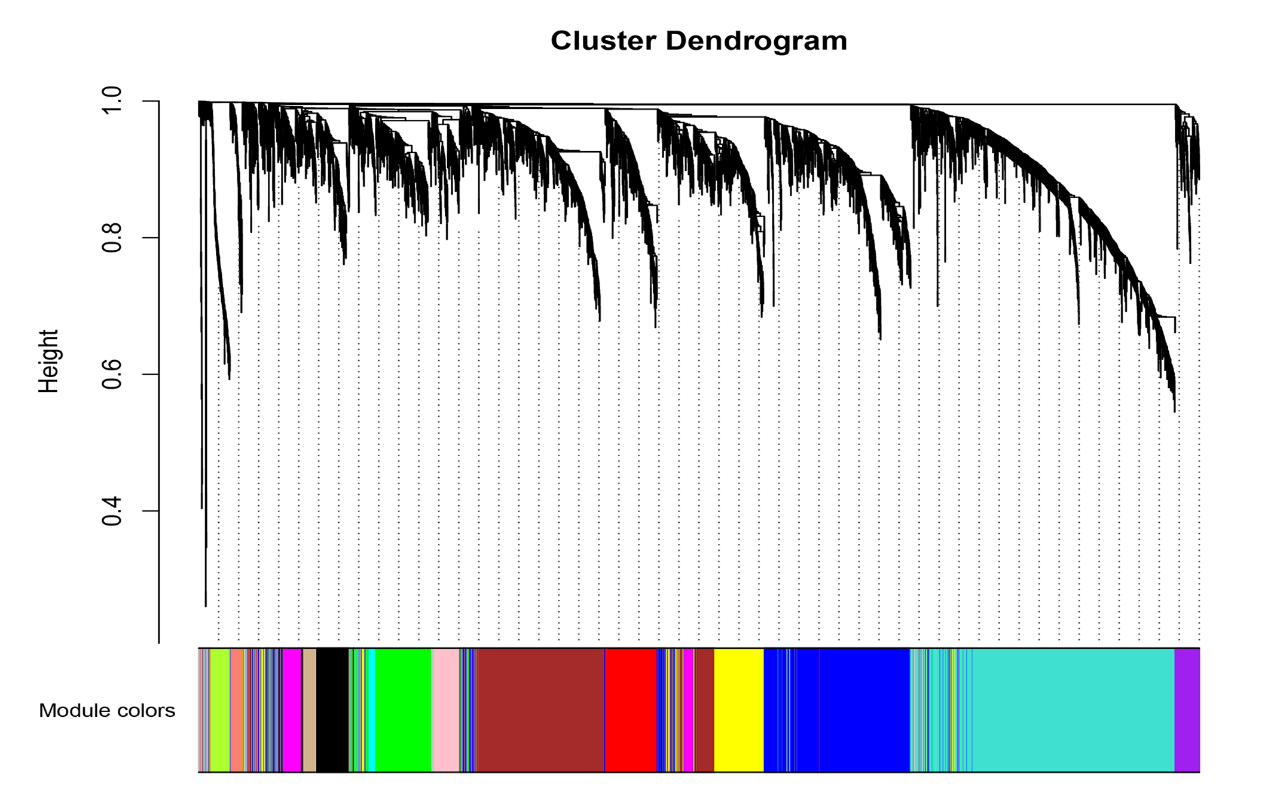


Figure S3: Clustering dendrogram of genes, with dissimilarity based on the topological overlap, together with assigned module colors.


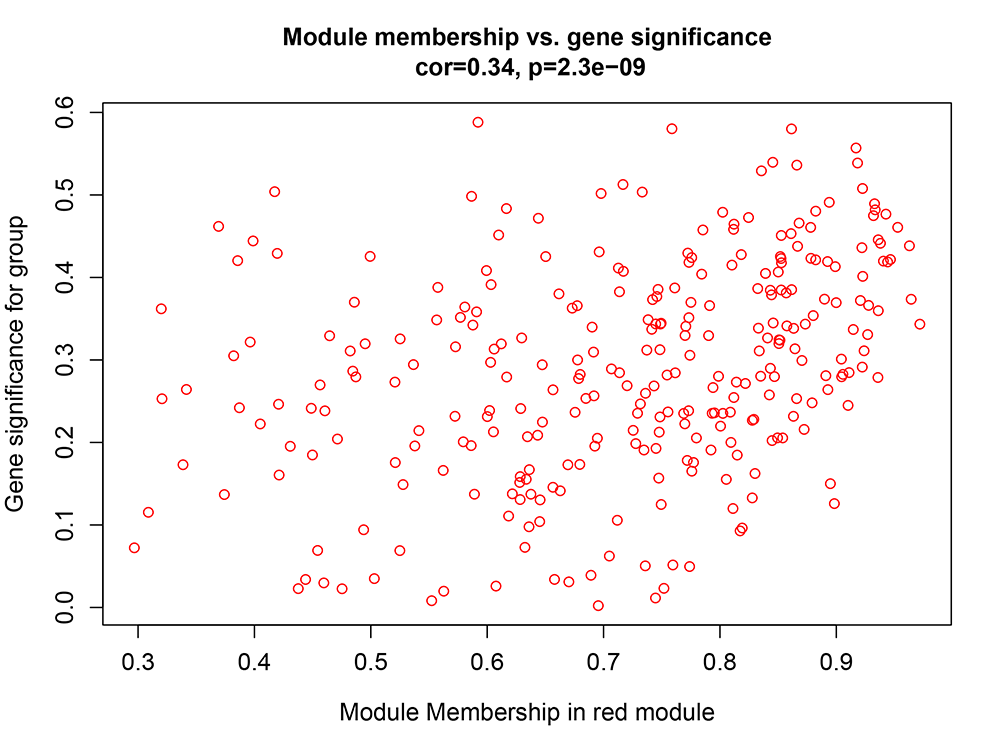


Figure S4: A scatterplot of Gene Significance (GS) for eosinophilic vs. Module Membership (MM) in the red module.


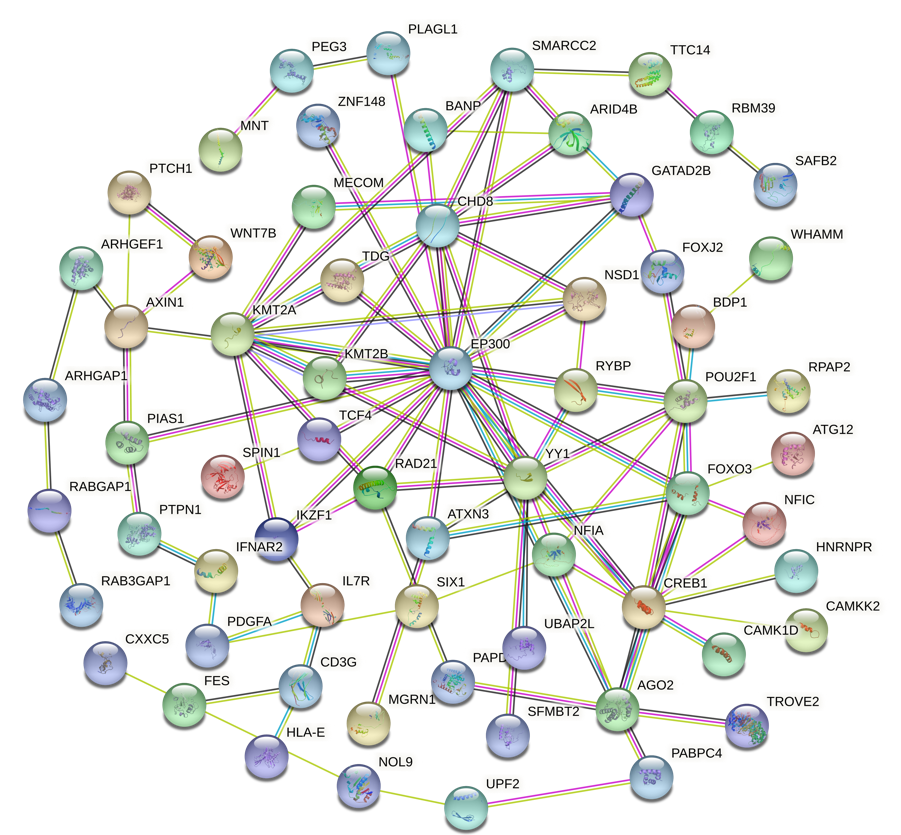


Figure S5a: The PPI network in the black modules


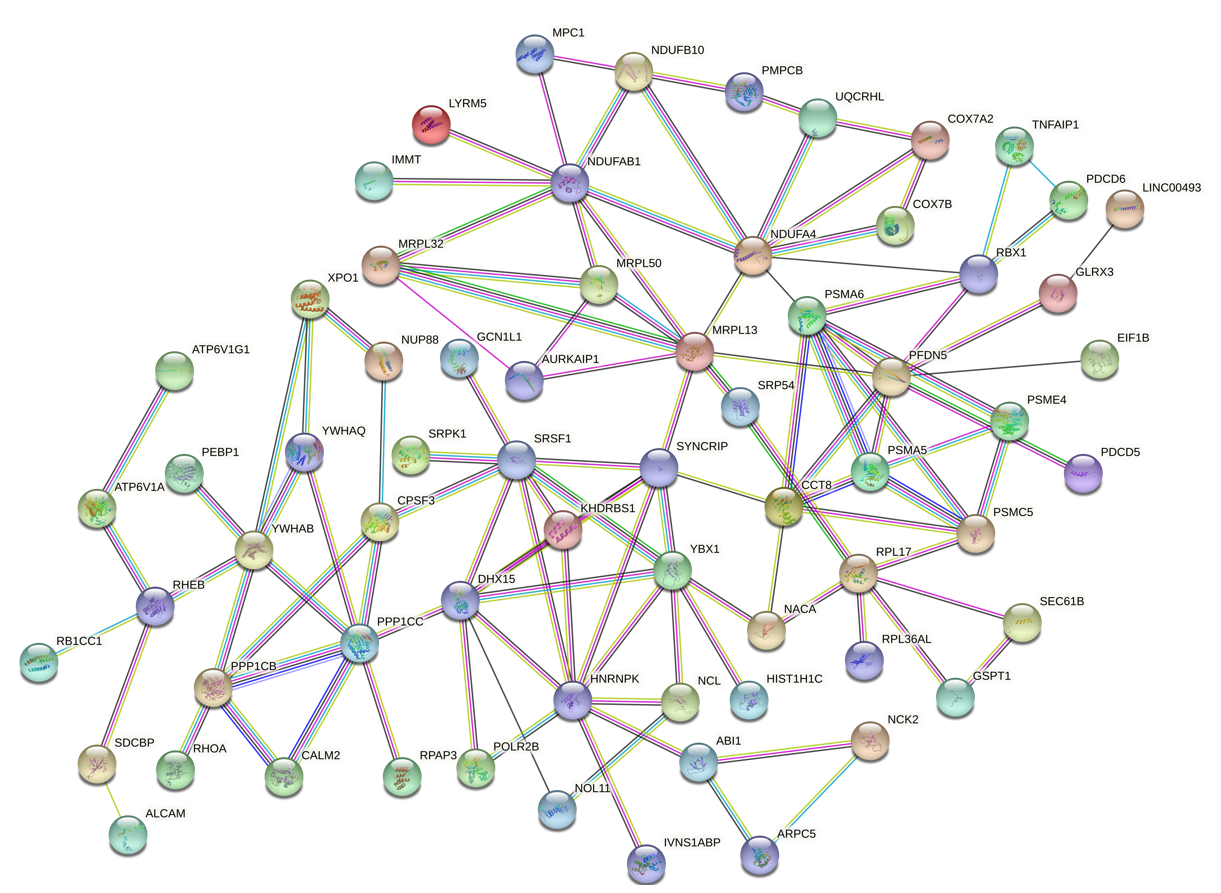


Figure S5c: The PPI network in the yellow module


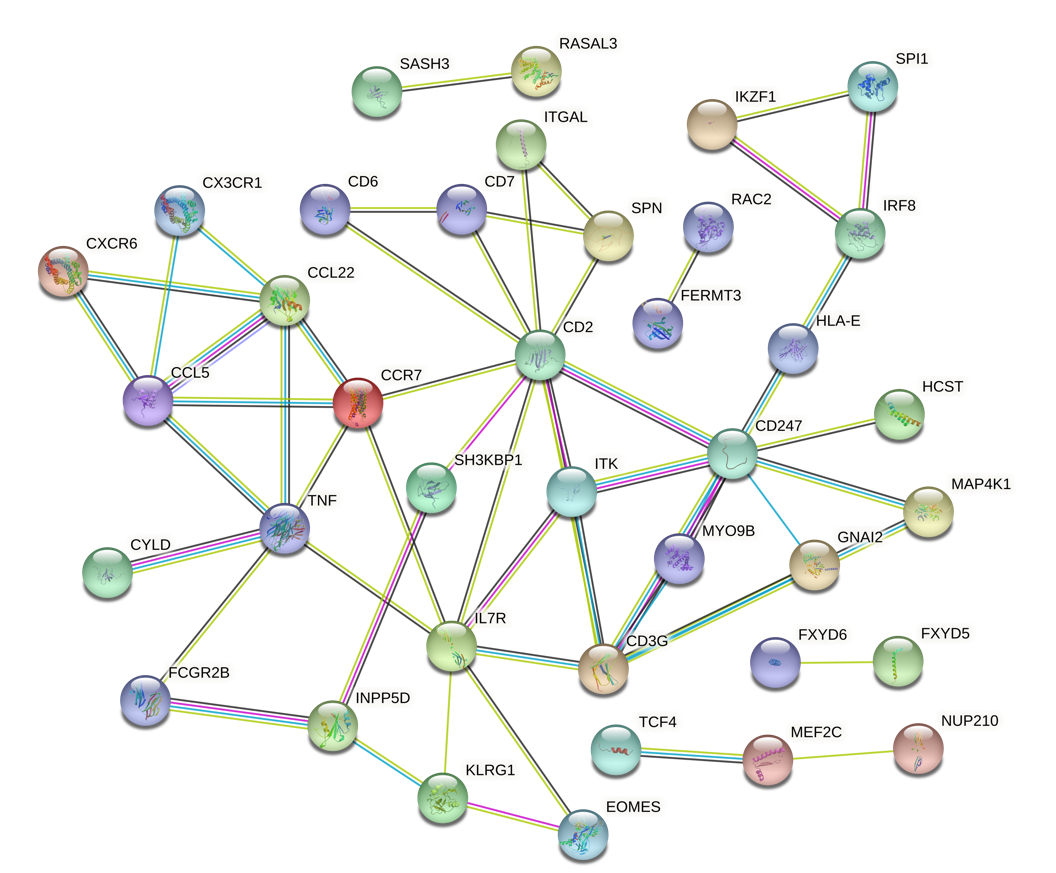


Figure S5b: The PPI network in the red modules


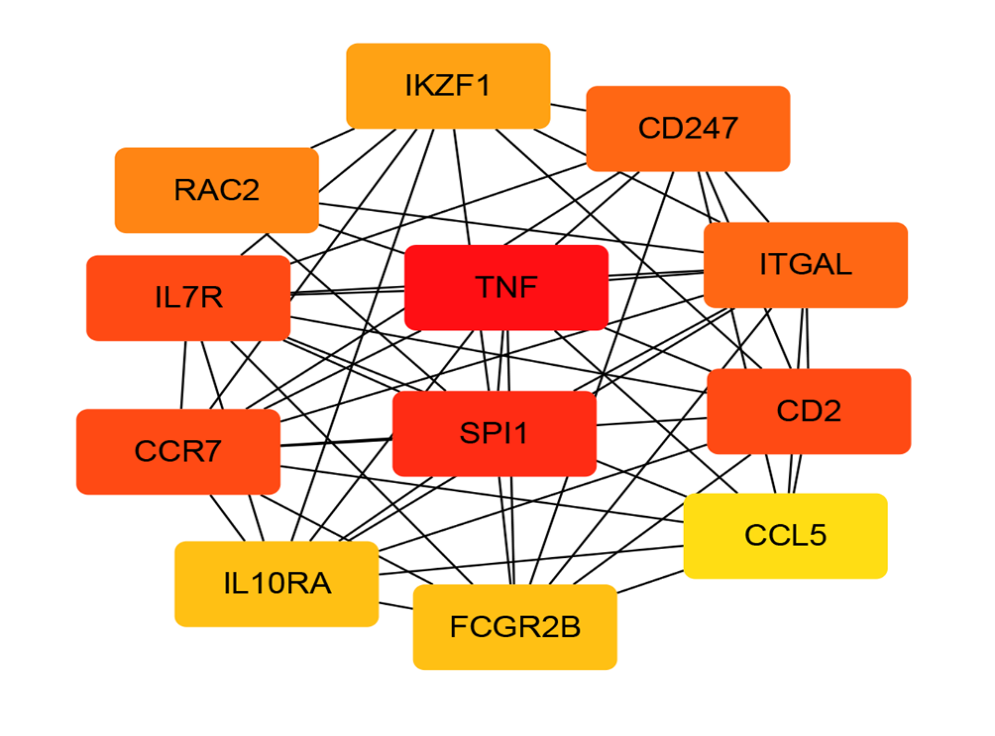


Figure S6: The top 12 genes in the red module. The darker the color, the higher the connectivity of the gene with other genes. The lighter the color, the less connected the gene is to the other genes.


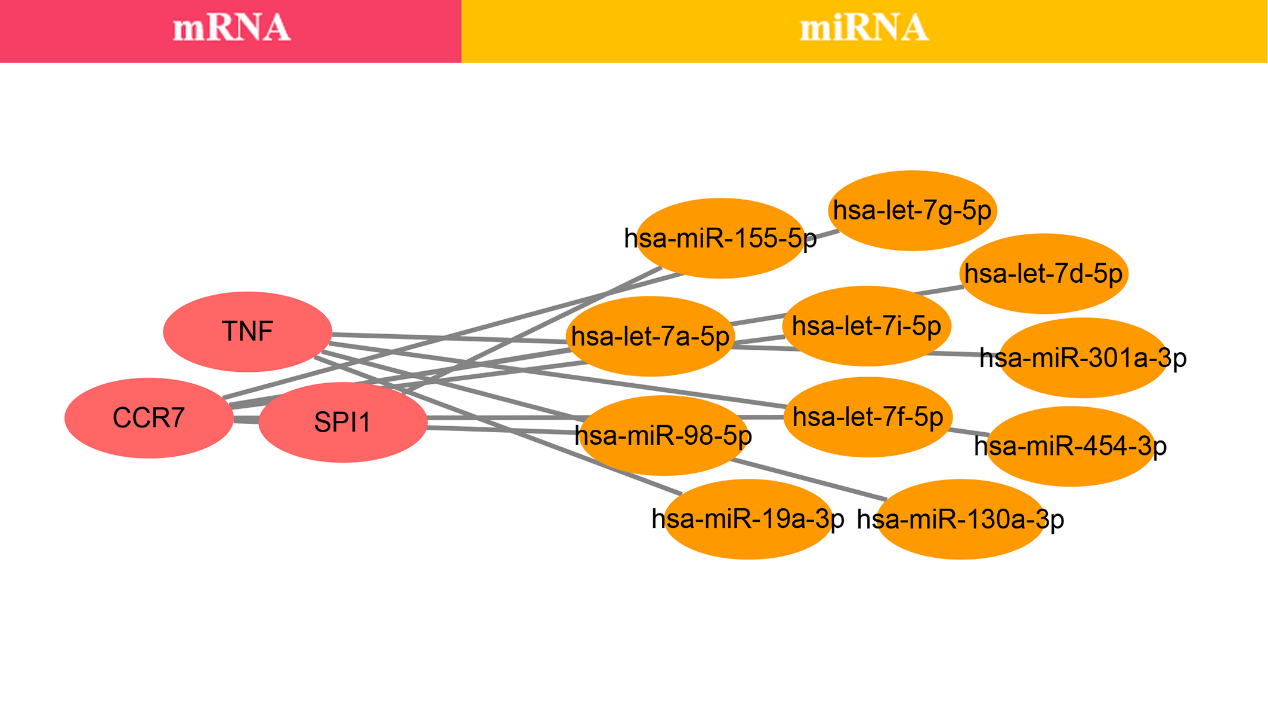


Figure S7: The miRNA-mRNA ceRNA network of the top 12 genes in the red modules


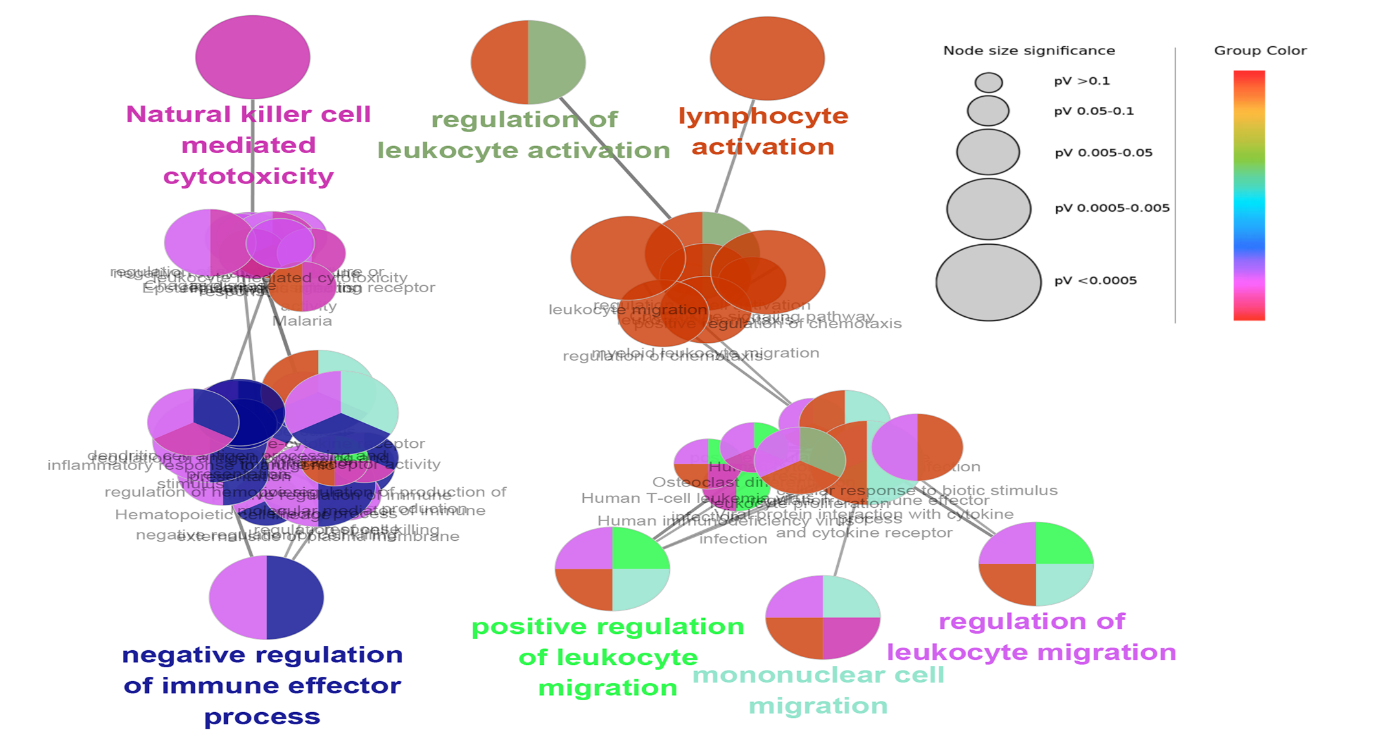


Figure S8: Functional enrichment of top 12 genes in the red modules. The signal pathways were discovered by enrichment analysis into groups based on functional connection, the same group was colored the same color, and the labels of each group of the most essential terms were color-coded.

| Table S1: The characteristics of participants in the GSE143303 | | |
| --- | --- | --- |
| Characteristic | HC(n=13) | SEA(n=22) |
| Age(years), mean (SD) | 45 (13) | 58 (13)* |
| Sex, female n (%) | 6 (46) | 12 (55) |
| Atopy, n (%) | 1 (8) | 13 (54) |
| BMI (kg/m2), mean (SD) | 31 (n = 1) | 30.1 (6.4) (n = 18) |
| Age of onset (%) |  |  |
| Childhood | N/A | 6/20 (30) |
| Adult | N/A | 14/20 (70) |
| FEV1%predicted, mean (SD) | 101.4 (10.7) | 74.3 (18.1)* |
| FVC %predicted, mean (SD) | 104.2 (9.9) | 88.5 (16.7) |
| FEV1/FVC, mean (SD) | 79.8 (3.9) | 66.8 (10.9)* |
| Former smoker, n (%) | 2 (15) | 5 (23) |
| Pack years, median (Q1, Q3) | 5 (5, 5) | 10 (5, 20) |
| ACQ score, mean (SD) | N/A | 2.0 (1.2) (n = 20) |
| ICS dose µg/day, median (Q1, Q3) | N/A | 1600 (800, 2000) |
| OCS use (%) | N/A | 5 (23) |
| OCS dose µg/day, median (Q1, Q3) | N/A | 10 (7.5, 10) |
| OCS courses, past 12 months, median (Q1, Q3) | N/A | 2 (1, 4) |
| HC: healthy control; SEA: severe eosinophilic asthma; ACQ: Asthma Control Questionnaire; ICS: inhaled corticosteroid; N/A: not applicable; OCS: oral corticosteroid; *: *P* < 0.008 versus HC. | | |

| Table S2: The characteristics of participants in the GSE143303 | | | |
| --- | --- | --- | --- |
| Characteristic | HC(n=13) | SNA(n=9) | SPA(n=16) |
| Age(years), mean (SD) | 45 (13) | 62(12)* | 55(14) |
| Sex, female n (%) | 6 (46) | 7(78) | 11(69) |
| Atopy, n (%) | 1 (8) | 6(67) | 6(40)(n=15) |
| BMI (kg/m2), mean (SD) | 31 (n = 1) | 24.4(2.6)(n=5) | 30.8(6.4)(n=12) |
| Age of onset (%) |  |  |  |
| Childhood | N/A | 5/7(71) | 8/14(57) |
| Adult | N/A | 2/7(29) | 6/14(43) |
| FEV1%predicted, mean (SD) | 101.4 (10.7) | 75.3 (14.1)* | 73.5(26.9)* |
| FVC %predicted, mean (SD) | 104.2 (9.9) | 97.4(19.3) | 91.9(19.4) |
| FEV1/FVC, mean (SD) | 79.8 (3.9) | 64.5(5.8)* | 64.5(17)* |
| Former smoker, n (%) | 2 (15) | 1(11) | 3(19) |
| Pack years, median (Q1, Q3) | 5 (5, 5) | 10(10,10) | 12.5(5,20) |
| ACQ score, mean (SD) | N/A | 1.4(1.3)(n=7) | 2.3(0.9)(n=15) |
| ICS dose µg/day, median (Q1, Q3) | N/A | 2000(1600,2000) | 2000(1300,2000) |
| OCS use (%) | N/A | 1(11) | 1(6) |
| OCS dose µg/day, median (Q1, Q3) | N/A | 15(15,15) | 15(15,15) |
| OCS courses, past 12 months, median (Q1, Q3) | N/A | 2(1,2) | 2.5(1,4.5) |
| HC: healthy control; SNA: severe neutrophilic asthma; SPA: severe paucigranulocytic asthma; ACQ: Asthma Control Questionnaire; ICS: inhaled corticosteroid; N/A: not applicable; OCS: oral corticosteroid; *: *P* < 0.05 versus HC. | | | |

| Table S3: The characteristics of participants in the GSE147878 | | | | |
| --- | --- | --- | --- | --- |
| characteristics | | HC(n=13) | severe asthma(n=42) | *P* value |
| age |  | 45.31±12.82 | 57.21±12.05 | 0.00* |
| gender | male | 7(53.85%) | 18(42.86%) | 0.49 |
|  | female | 6(46.15%) | 24(57.14%) |  |
| OCS | no | 13(100.00%) | 35((83.33%) | 0.12 |
|  | yes | 0(0.00%) | 7(16.67%) |  |
| smoking | nonsmoker | 11(84.62%) | 32(76.19%) | 0.52 |
|  | former smoker | 2(15.38%) | 10(23.81%) |  |
| atopy | no | 12(92.31%) | 18(42.86%) | 0.00* |
|  | yes | 1(7.69%) | 24(57.14%) |  |
| prednisone | no | 13(100.00%) | 36(85.71%) | 0.15 |
|  | yes | 0(0.00%) | 6(14.29%) |  |
| **P*<0.05; OCS: oral corticosteroid; HC: health control | | | | |

| Table S4: The characteristics of the participants in the GSE137268 | | | | |
| --- | --- | --- | --- | --- |
| characteristics | | HC(n=15) | Non-severe eosinophilic asthma(n=13) | *P* value |
| age |  | 44.6±21.44 | 55.38±19.22 | 0.18 |
| gender | male | 7(46.67%) | 6(46.15%) | 0.98 |
|  | female | 8(53.33%) | 7(53.85%) |  |

HC: health control

| Table S5: the hub genes after adjusted age the red module | | | | | | |
| --- | --- | --- | --- | --- | --- | --- |
| Hub genes | B | Standard error | Wald | *P* value | OR | 95%CI |
| TNF | -0.83 | 0.53 | 2.46 | 0.12 | 0.44 | 0.16-1.23 |
| SPI1 | -0.12 | 0.14 | 0.69 | 0.41 | 0.89 | 0.68-1.17 |
| CCR7 | -0.16 | 0.11 | 1.83 | 0.18 | 0.86 | 0.68-1.07 |
| IL7R | -0.22 | 0.13 | 2.93 | 0.09 | 0.80 | 0.62-1.03 |
| CD2 | -0.21 | 0.16 | 1.77 | 0.18 | 0.81 | 0.60-1.10 |
| ITGAL | -0.41 | 0.21 | 3.74 | 0.06 | 0.66 | 0.44-1.01 |
| CD247 | -0.43 | 0.20 | 4.62 | 0.07 | 0.65 | 0.44-0.96 |
| RAC2 | -0.04 | 0.05 | 0.64 | 0.42 | 0.96 | 0.87-1.06 |
| IKZF1 | -0.25 | 0.20 | 1.55 | 0.21 | 0.78 | 0.53-1.15 |
| IL10RA | -0.16 | 0.10 | 2.54 | 0.11 | 0.85 | 0.69-1.04 |
| FCGR2B | -0.42 | 0.33 | 1.67 | 0.20 | 0.66 | 0.35-1.24 |
| CCL5 | -0.13 | 0.08 | 2.49 | 0.11 | 0.88 | 0.75-1.03 |

| \| Table S6: the hub genes of the black module verified in GSE 143303 \| \| \| \| \| \| --- \| --- \| --- \| --- \| --- \| \| Hub genes \| HC(n=13) \| Severe non-eosinophilic asthma(n=23) \| t value \| *P* value \| \| EP300 \| 0.28±0.51 \| -0.21±0.46 \| 3.09 \| 0.00* \| \| YY1 \| 0.22±0.46 \| -0.11±0.37 \| 2.49 \| 0.02* \| \| CREB1 \| 0.43±0.39 \| -0.06±0.22 \| 4.88 \| 0.00* \| \| POU2F1 \| 0.15±0.30 \| -0.04±0.18 \| 2.51 \| 0.02* \| \| KMT2A \| 0.14±0.46 \| -0.18±0.41 \| 2.24 \| 0.03* \| \| CHD8 \| 0.16±0.40 \| -0.14±0.26 \| 2.80 \| 0.01* \| \| AGO2 \| 0.48±0.55 \| -0.08±0.35 \| 3.76 \| 0.00* \| \| FOXO3 \| 0.15±0.50 \| -0.05±0.47 \| 1.28 \| 0.21 \| \| RAD21 \| 0.34±0.47 \| -0.03±0.44 \| 2.49 \| 0.02* \| \| AXIN1 \| 0.20±0.26 \| -0.03±0.25 \| 2.69 \| 0.01* \| \| SMARCC2 \| 0.21±0.27 \| -0.07±0.20 \| 3.65 \| 0.00* \| \| GATAD2B \| 0.27±0.41 \| -0.12±0.23 \| 3.75 \| 0.00* \|   HC: health control; **P*<0.05  Table S7: the hub genes of the black module verified in GSE 147878 | | | | |
| --- | --- | --- | --- | --- | --- | --- | --- | --- | --- | --- | --- | --- | --- | --- | --- | --- | --- | --- | --- | --- | --- | --- | --- | --- | --- | --- | --- | --- | --- | --- | --- | --- | --- | --- | --- | --- | --- | --- | --- | --- | --- | --- | --- | --- | --- | --- | --- | --- | --- | --- | --- | --- | --- | --- | --- | --- | --- | --- | --- | --- | --- | --- | --- | --- | --- | --- | --- | --- | --- | --- | --- | --- | --- | --- |
| Hub genes | HC(n=13) | severe asthma(n=42) | t value | *P* value |
| EP300 | 8.60±0.25 | 8.12±0.34 | 4.71 | 0.00* |
| YY1 | 10.43±0.22 | 9.94±0.37 | 4.54 | 0.00* |
| CREB1 | 11.71±0.51 | 11.35±0.62 | 1.90 | 0.05* |
| POU2F1 | 8.44±0.24 | 8.22±0.17 | 3.56 | 0.00* |
| KMT2A | 8.20±0.21 | 7.83±0.30 | 4.00 | 0.00* |
| CHD8 | 10.13±0.16 | 9.68±0.29 | 5.26 | 0.00* |
| AGO2 | 9.93±0.26 | 9.16±0.34 | 7.49 | 0.00* |
| FOXO3 | 10.89±0.31 | 10.49±0.44 | 3.02 | 0.00* |
| RAD21 | 8.48±0.26 | 8.02±0.40 | 3.87 | 0.00* |
| AXIN1 | 8.89±0.17 | 8.65±0.23 | 3.49 | 0.00* |
| SMARCC2 | 9.59±0.30 | 8.94±0.42 | 5.15 | 0.00* |
| GATAD2B | 7.81±0.26 | 7.46±0.17 | 5.74 | 0.00* |

HC: health control; **P*<0.05

| Table S8: the hub genes of the black module verified in GSE 137268 | | | | |
| --- | --- | --- | --- | --- |
| Hub genes | HC(n=15) | Non-severe eosinophilic asthma (n=13) | t value | *P* value |
| EP300 | 0.13±0.22 | -0.07±0.54 | 1.31 | 0.20 |
| YY1 | 0.10±0.15 | -0.02±0.32 | 1.21 | 0.24 |
| CREB1 | -0.04±0.17 | 0.02±0.30 | -0.66 | 0.52 |
| POU2F1 | 0.10±0.31 | 0.17±0.47 | -0.43 | 0.67 |
| KMT2A | 0.09±0.18 | -0.10±0.30 | 2.09 | 0.05* |
| CHD8 | 0.16±0.30 | 0.04±0.29 | 1.07 | 0.29 |
| AGO2 | -0.14±0.54 | 0.18±0.72 | -1.34 | 0.19 |
| FOXO3 | -0.08±0.18 | 0.20±0.35 | -2.71 | 0.01* |
| RAD21 | -0.07±0.32 | 0.02±0.43 | -0.62 | 0.54 |
| AXIN1 | -0.04±0.25 | 0.22±0.39 | -2.15 | 0.04* |
| SMARCC2 | 0.22±0.29 | 0.10±0.23 | 1.12 | 0.27 |
| GATAD2B | -0.03±0.36 | -0.06±0.40 | 0.19 | 0.85 |

HC: health control; **P*<0.05

| Table S9 the hub genes of the yellow module verified in GSE 143303 | | | | |
| --- | --- | --- | --- | --- |
| Hub genes | HC(n=13) | severe non-eosinophilic asthma (n=23) | t value | *P* value |
| SRSF1 | -0.24±0.35 | 0.07±0.24 | -3.27 | 0.00* |
| PFDN5 | -0.20±0.29 | 0.07±0.20 | -3.34 | 0.00* |
| MRPL13 | -0.25±0.49 | 0.12±0.34 | -2.72 | 0.01* |
| SYNCRIP | -0.13±0.34 | 0.15±0.32 | -2.54 | 0.02* |
| NDUFAB1 | -0.18±0.34 | 0.15±0.32 | -2.87 | 0.01* |
| POLR2B | -0.16±0.24 | 0.13±0.21 | -3.84 | 0.00* |
| PSMA6 | -0.33±0.40 | 0.16±0.28 | -4.46 | 0.00* |
| DHX15 | -0.19±0.39 | 0.18±0.29 | -3.40 | 0.00* |
| PPP1CC | -0.19±0.50 | 0.19±0.36 | -2.76 | 0.01* |
| RPL17 | -0.08±0.30 | 0.08±0.26 | -1.75 | 0.09 |
| HNRNPK | 0.05±0.15 | 0.02±0.13 | 0.61 | 0.55 |
| CCT8 | -0.22±0.32 | 0.07±0.22 | -3.32 | 0.00* |

HC: health control; **P*<0.05

| Table S10: the hub genes of the yellow module verified in GSE 147878 | | | | |
| --- | --- | --- | --- | --- |
| Hub genes | HC(n=13) | severe asthma(n=42) | t value | *P* value |
| SRSF1 | 8.83±0.33 | 9.15±0.31 | -3.26 | 0.00* |
| PFDN5 | 10.50±0.24 | 10.84±0.28 | -3.98 | 0.00* |
| MRPL13 | 7.91±0.35 | 8.29±0.24 | -4.43 | 0.00* |
| SYNCRIP | 7.68±0.29 | 7.91±0.25 | -2.78 | 0.01* |
| NDUFAB1 | 7.49±0.15 | 7.82±0.24 | -4.63 | 0.00* |
| POLR2B | 7.85±0.15 | 8.07±0.21 | -3.41 | 0.00* |
| PSMA6 | 10.26±0.40 | 10.71±0.21 | -5.37 | 0.00* |
| DHX15 | 8.80±0.35 | 9.16±0.28 | -3.86 | 0.00* |
| PPP1CC | 8.07±0.46 | 8.47±0.34 | -3.41 | 0.00* |
| RPL17 | 13.35±0.16 | 13.31±0.15 | 0.91 | 0.37 |
| HNRNPK | 9.90±0.68 | 9.790.85 | 0.42 | 0.68 |
| CCT8 | 9.83±0.27 | 10.21±0.22 | -5.21 | 0.00* |

HC: health control; **P*<0.05

| Table S11: the hub genes of the yellow module verified in GSE 137268 | | | | |
| --- | --- | --- | --- | --- |
| Hub genes | HC (n=15) | Non-severe eosinophilic asthma (n=13) | t value | *P* value |
| SRSF1 | 0.06±0.25 | -0.06±0.21 | 1.40 | 0.17 |
| PFDN5 | -0.01±0.33 | 0.08±0.50 | -0.60 | 0.55 |
| MRPL13 | -0.08±0.37 | -0.18±0.41 | 0.70 | 0.49 |
| SYNCRIP | -0.01±0.33 | -0.04±0.32 | 0.20 | 0.85 |
| NNDUFAB1 | -0.02±0.35 | -0.12±0.53 | 0.59 | 0.56 |
| POLR2B | -0.04±0.19 | 0.07±0.16 | -1.66 | 0.11 |
| PSMA6 | -0.19±0.28 | -0.17±0.42 | -0.21 | 0.83 |
| DHX15 | 0.03±0.22 | -0.09±0.28 | 1.28 | 0.21 |
| PPP1CC | -0.01±0.25 | -0.11±0.43 | 0.70 | 0.49 |
| RPL17 | -0.14±0.24 | 0.06±0.38 | -1.66 | 0.11 |
| HNRNPK | -0.23±0.27 | 0.01±0.47 | -1.69 | 0.10 |
| CCT8 | 0.00±0.28 | -0.04±0.33 | 0.39 | 0.70 |

HC: health control; **P*<0.05

| Table S12: the hub genes of the red module verified in GSE 143303 | | | | |
| --- | --- | --- | --- | --- |
| Hub genes | HC(n=13) | severe non-eosinophilic asthma (n=23) | t value | *P* value |
| TNF | 0.21±0.35 | 0.14±0.40 | 0.51 | 0.62 |
| SPI1 | 0.15±0.44 | 0.07±0.49 | 0.54 | 0.59 |
| CCR7 | 0.45±0.61 | 0.33±0.61 | 0.57 | 0.57 |
| IL7R | 0.38±0.80 | 0.15±0.95 | -0.43 | 0.67 |
| CD2 | 0.51±0.69 | 0.20±0.67 | 1.36 | 0.18 |
| RAC2 | 0.28±0.66 | 0.05±0.80 | 0.92 | 0.36 |
| IKZF1 | 0.25±0.35 | 0.26±0.62 | -0.02 | 0.98 |
| IL10RA | 0.18±0.56 | 0.26±0.64 | -0.37 | 0.72 |
| FCGR2B | 0.15±0.28 | 0.09±0.36 | 0.53 | 0.60 |
| CCL5 | 0.64±0.75 | 0.16±0.86 | 1.75 | 0.09 |

HC: health control;
